# ^1^H NMR Profiling of the Venom from *Hylesia continua*: Implications of Small Molecules for Lepidopterism

**DOI:** 10.3390/toxins15020101

**Published:** 2023-01-20

**Authors:** Nemesio Villa-Ruano, Elvia Becerra-Martínez, José María Cunill-Flores, Jorge Ariel Torres-Castillo, Guillermo M. Horta-Valerdi, Yesenia Pacheco-Hernández

**Affiliations:** 1 CONACyT-Centro Universitario de Vinculación y Transferencia de Tecnología, Benemérita Universidad Autónoma de Puebla, Puebla 72570, Mexico; 2 Centro de Nanociencias y Micro y Nanotecnologías, Instituto Politécnico Nacional, Av. Luis Enrique Erro s/n, Unidad Profesional Adolfo López Mateos, Mexico City 07738, Mexico; 3 Ingeniería en Biotecnología, Universidad Politécnica Metropolitana de Puebla, Popocatépetl s/n, Reserva Territorial Atlixcáyotl, Tres Cerritos, Puebla 72480, Mexico; 4 Instituto de Ecología Aplicada, Universidad Autónoma de Tamaulipas, Ave. División del Golfo 356, Colonia Libertad, Ciudad Victoria 87019, Mexico

**Keywords:** *Hylesia continua*, lepidopterism, inflammation, pain-inducing, small molecules

## Abstract

Lepidopterism caused by caterpillar contact is considered a public health problem around the world. The local and systemic responses of this pathology include short- and long-term inflammatory events. Although the proteolytic activity of the venoms from caterpillars is strongly associated with an inflammatory response in humans and murine models, fast and acute symptoms such as a burning sensation, itching, and pain should be related to the presence of low-weight hydrophilic molecules which easily influence cell metabolism. This investigation reports on the ^1^H-Nuclear Magnetic Resonance (NMR) profiling of the venom from the larva of *Hylesia continua*, a caterpillar linked to frequent cases of lepidopterism in the northern highlands of Puebla, Mexico. According to one-dimensional (1D) and two-dimensional (2D) NMR data, the venom of *H. continua* contained 19 compounds with proven pain-inducing activity (i.e., acetic acid, lactic acid, formic acid, succinic acid, 2-hydroxyglutaric acid, ethanol, and glutamate), inflammatory activity (i.e., cadaverine, putrescine, and acetoin), as well as natural immunosuppressive activity (i.e., O-phosphocholine and urocanic acid). The levels of the 19 compounds were calculated using quantitative-NMR (qNMR) and extensively discussed on the basis of their toxic properties which partially explain typical symptoms of lepidopterism caused by the larvae of *H. continua*. To the best of our knowledge, this is the first investigation reporting a complex mixture of small molecules with inflammatory properties dissolved in the venom of a lepidopteran larva.

## 1. Introduction

The term “Lepidopterism” is related to cutaneous and systemic reactions (dermatitis) from physical contact with organisms of the order Lepidoptera including caterpillars, moths, or butterflies [1]. The primary clinical manifestations of lepidopterism include stinging, itching, hypersensitivity reactions, lonomism, and rare cases of oropharyngeal manifestations that depend on the type of venom injected by the causal agent as well as on the sensitivity of the affected person [2]. Although lepidopterism is not lethal for humans, it is considered a public health problem in North, Central, and South America since thousands of cases are recorded per year, being more frequent in children younger than 6 years and farmers [1,2,3,4]. In the 21st century, envenomation by caterpillars is still clinically challenging owing to their potential to provoke a diverse array of symptoms. Treatments are mainly supportive and not efficient since the only specific treatment on the market is the Lonomia antivenom. Such antivenom is used to address the intoxication by the extremely venomous *Lonomia obliqua* (Saturniidae) also known as Taturana or fire caterpillar in southern Brazil [5,6]. It is believed that the increase in lepidopterism incidence is caused by several environmental changes associated with anthropogenic activity including those produced by climate change [7]. the incidence of this affectation is related to the colonization and life cycle development of lepidopteran species in diverse monocultures that cause devastating losses [1,2,3,4,5,6]. Dermatitis produced by these insects is usually resistant to all therapeutic treatments since their venom is considered a complex cocktail of inflammatory molecules [1,2,3,4,5,6,7].

The moths of the *Hylesia* genus, especially those of *H. metabus* and *H. nigricans*, are the most common species associated with lepidopterism because of their ample distribution in America and Europe [8,9,10,11,12,13,14]. Nevertheless, *H. continua* has also been associated with severe cases of lepidopterism which include erythematous lesions, intense itching, and intermittent fever [14]. The larvae from this species are known with the generic name of “tachichinas” or “cuatechicalillos” in the northern highlands of Puebla, Mexico. The insects frequently trigger cases of lepidopterism in farmers during maize harvesting between August and September every year. Nevertheless, some cases are reported throughout the year.

Current efforts to characterize the chemical content of the venoms form different *Hylesia* species are few and controversial since the chemical composition of the venom from their larvae and moths did not reveal the presence of common inflammatory molecules such as histamine, formic acid, or acetylcholine as for plant stinging hair’s fluid [15]. Previous studies reported that the venom from the moths of *H. metabus* contains serine proteases apparently associated with a long-term urticating effect in a murine model [16]. Interestingly, the in situ location of the proteolytic toxin from *H. metabus* was identified in the inner parts of the urticating setae, and its inflammatory effect was endorsed in distinct murine models by hystopathological analysis [17]. Recent studies sustain that the protease profile of the venom from caterpillars and female moths of *Hylesia* sp. substantially varies [18]. The content of serpins and serine proteases was different in the female moth venom which showed higher caseinolytic activity than in caterpillar venom. The latter venom showed high capacity to hydrolyze fibrinogen and gelatin [18]. In addition, the female venom displayed a dose-dependent procoagulant effect [18].

The study of venoms from moths and caterpillars has been addressed from diverse analytical techniques. Traditionally, sodium dodecyl sulfate polyacrylamide gel electrophoresis (SDS-PAGE) and high-performance liquid chromatography (HPLC) were used to visualize, separate, and quantify protein fractions. Although these methods are effective for visualizing the complexity of the venomous mixture, low abundant components are often overlooked [5]. This limitation has sparked the development of refined methods based in proteomics, transcriptomics, and genomics to know the venomous composition in detail. Nevertheless, to the best of our knowledge, there is not any approach on the nuclear magnetic resonance (NMR) profiling applied for the same aim. As is known, NMR is the most powerful technique used to assign the metabolite identity of individual compounds and mixtures of them. Although NMR is less sensitive than chromatographic methods coupled to mass spectrometry, modern high-definition apparatus may give a solid overview on the compounds dissolved in biological samples without fine separation [19]. This fact makes the NMR method highly robust and reliable to determine and quantify small molecules of diverse chemical nature.

On the bases of the latter arguments, this work aimed to obtain the ^1^H NMR profiling of the venom from *H. continua* as one of the most frequent causal agents of lepidopterism in the northern highlands of Puebla, Mexico.

## 2. Results

### 2.1. Morphological and Molecular Features of Hylesia continua

**Figure 1:**
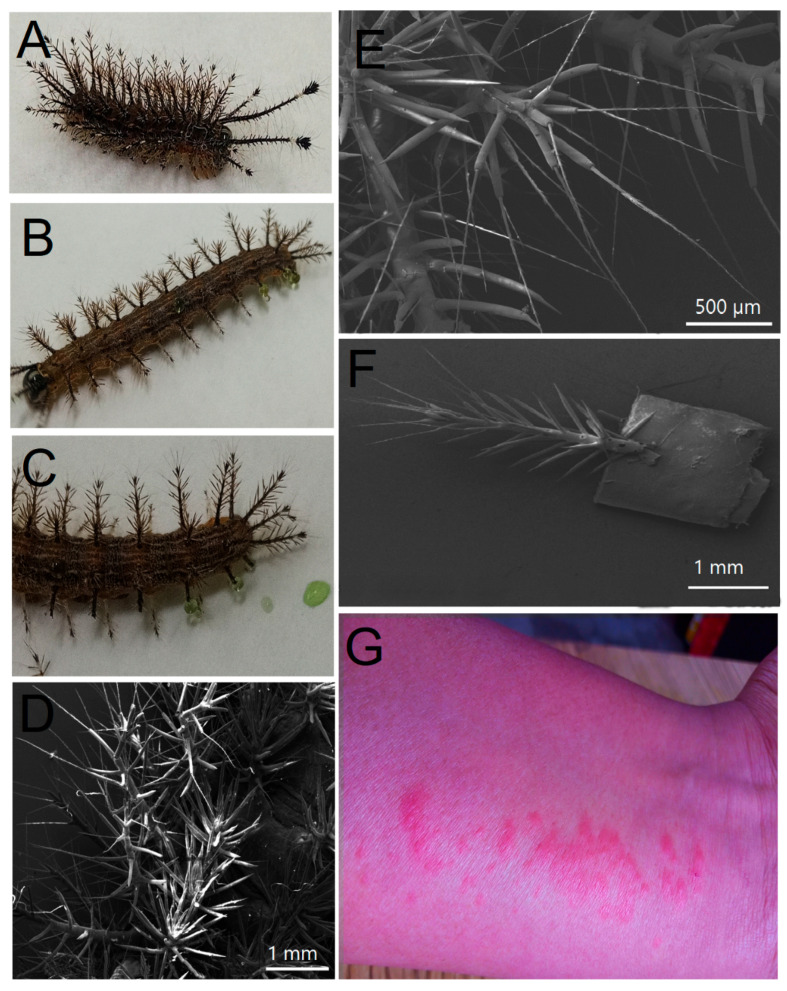
Morphological features of *Hylesia continua* as a causal agent of lepidopterism in the northern highlands of Puebla, Mexico. (**A**,**B**) Lateral views of *H. continua* showing urticating setae, black head capsule and head horns. (**C**) Excised lateral setae excreting venom drops. (**D**) SEM abdominal view showing rows containing composed setae with terminal bristles (white scale bar is equivalent to 1 mm). (**E**) SEM view of fine urticating hairs adhered to composed setae (white scale bar is equivalent to 500 µm). (**F**) SEM view of single lateral seta showing urticating bristles (white scale bar is equivalent to 1 mm). (**G**) Urticaria produced by direct contact of *H. continua* setae with human skin.

The larvae collected in maize plots from the municipality of Yaonahuac, Puebla, México, presented bodies of brown color with 6 rows containing 12 composed setae (1–2 mm) with white terminal bristles and prominent spine-horns (5–6 mm) in the head capsule (2) and anal plate (5). There are four pairs of abdominal prolegs, one pair of anal prolegs, and two pairs of thoracic legs (Figure 1A–F). The larvae showed intercalated strips of white, brown, and orange color, a black head capsule, and a black anal plate which gives the dorsal surface a brown-caramel appearance at first glance (Figure 1A–F). These organisms had average measurements of 2.5–3 cm in length and 3–5 mm in width, whereas the exudate from setae (obtained after mechanical damage) had an emerald green color (Figure 1C). According to the features of the latter, the collected samples belonged to the third instar of *Hylesia continua* [20]. The physical contact of the spines with human skin resulted in acute dermatitis according to medical records from the rural health center of Yaonahuac, Puebla, Mexico (Figure 1G) [21]. The spine exudate was subsequently collected and analyzed via one-dimensional (1D) and two-dimensional (2D) NMR.

Analysis of the cytochrome oxidase subunit 1 (COI) gene (Sequences S1–S2) confirmed 97% identity with certified vouchers of *H. continua* linked to the accessions GU146591.1, MK612337.1, MK612335.1, MK612334.1, MK612333.1, and MK612332.1 reported by Rabl et al. [22], which were stored at the nucleotide databank of the National Center for Biotechnology Information (NCBI). The sequence also showed homology with 30 other records of *H. continua* deposited in the same electronic source. The morphological and molecular features confirmed the identity of the studied larvae as *H. continua*.

### 2.2. ^1^H NMR Profiling of the Venom from Hylesia continua

Nineteen compounds were identified in the venom of *H. continua* by 1D and 2D NMR experiments (Table 1). Six amino acids including alanine, glutamate, glycine, phenylalanine, proline, and histidine, which is biosynthetically related to histamine and urocanic acid, were detected. Interestingly, known organic acids associated with the inflammatory response or considered pain-inducing toxins were determined. These were acetic acid, formic acid, lactic acid, succinic acid, 2-hydroxyglutaric acid, and urocanic acid. The ^1^H NMR spectrum of the venom from *H. continua* clearly showed a high abundance of acetic acid, succinic acid, and phosphocholine (Figure S1). On the other hand, the spectral region of 0.9–4.5 ppm contained protons belonging to amino acids and organic acids. In this particular region, 13 metabolites were fully identified (Figure S1). Conversely, the spectral region of 6.0–9.5 ppm was linked to the presence of phenolic compounds (Figure S1).

**Figure 2:**
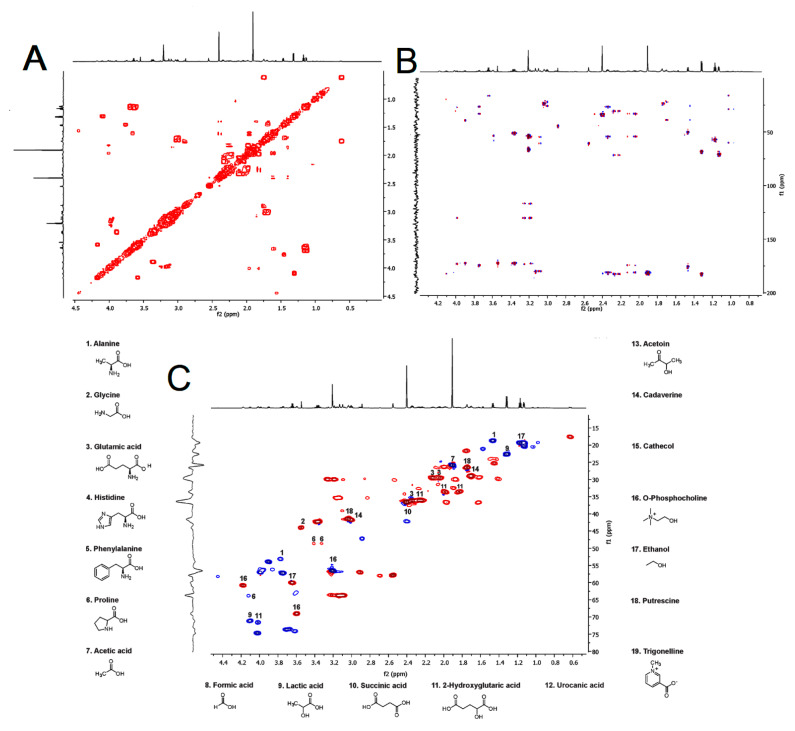
Correlated spectroscopy spectrum (**A**), heteronuclear multiple bond correlation spectrum (**B**), and heteronuclear single quantum coherence spectrum (**C**) of the venom from *Hylesia continua*.

Interestingly, compounds with immunomodulatory activity were also determined. Among these compounds, biogenic amines such as cadaverine, putrescine, as well as ethanol, acetoin, urocanic acid, phosphocholine, catechol, and trigonelline were found in the venom of *H. continua*. The identity of these metabolites was endorsed by correlated spectroscopy (COSY) which was used to estimate the proton–proton correlation in the ^1^H NMR spectrum (Figure 2A), whereas the spectra from heteronuclear single quantum coherence spectrum (HSQC) and heteronuclear multiple bond correlation (HMBC) revealed the heteronuclear correlation (^1^H and ^13^C) among single bonds and multiple bonds in the metabolites analyzed (Figure 2B,C). The NMR data were compared with those of the Human Metabolome Database (HMDB).

The results of the qNMR analysis showed that the main small molecule dissolved in the venom from *H. continua* was acetic acid (28 mM), followed by succinic acid and ethanol (>9 mM) (Table 2). Levels over 6 mM were estimated for lactic acid and 2-hydroxyglutaric acid, whereas those of glutamic acid were higher than 5 mM. The molar levels of phosphocholine, alanine, and glycine were similar (~3 Mm). A comparable trend was observed for proline and histidine (~1 mM). Interestingly, the levels of biogenic amines (putrescine and cadaverine) were quite close (~1 mM). Other compounds such as phenylalanine, formic acid urocanic acid, acetoin, catechol, and trigonelline were found in concentrations lower than 1 mM. These results confirmed that the venom of *H. continua* contains a complex cocktail of small molecules, most of them associated with an inflammatory response.

## 3. Discussion

To the best of our knowledge, there is little information on the determination of small molecules from any animal venom via high-throughput NMR and none performed on the venom of caterpillars using the same analytical technique. Previous studies based in ^1^H NMR profiling revealed that the venom of spider species contain polyamines (spermidine), common neurotransmitters (gamma-aminobutyric acid and choline), amino acid derivatives, as well as sulfated nucleosides [23]. In the same context, NMR has been used to screen the metabolic changes of honeybee bodies in different seasons [24]. This technique was successfully applied to determine punctual fluctuations in the content of carbohydrates, amino acids, and choline-containing compounds in insect body parts [24].

Current research on caterpillar’s venom has mainly been focused on the characterization of proteases that induce an aggressive and long-term inflammatory response in human skin [5,16,17,18]. The possible synergy of pain-inducing small molecules on the inflammation process has basically been ignored [5,16,17,18]. The venom of caterpillars usually causes an immediate and acute pain or burning sensation which cannot necessarily be derived from the damage of protease activity. The presence of several pain-inducing molecules including the classic formate excreted by ants or other organic acids, such as acetic and lactic acids, may play a substantial role in the early symptomatology earned by some species of caterpillars such as *H. continua* [25].

Acetic acid is basically a corrosive substance which is destined for cosmetic use (peeling) or for antiseptic aims; however, the intradermic exposition of the organic acid may cause ulcers and a burning sensation since this compound triggers local inflammation [26]. The use of acetic acid as a therapeutic agent seems controversial because of its acute pain effect in animal models [27]. Other known organic acids with pain-inducing properties, such as formic acid, were also detected in the venom of *H. continua*. This compound has been reported as the most abundant toxin found in several ant species [28]. It is widely known that the accumulation of lactic acid in skeletal muscle produces acute pain; however, not all tissues are able to efficiently metabolize this metabolite. The effect of lactate may vary depending on the metabolic status and cell type. This organic acid modulates signaling pathways releasing cytokines, chemokines, adhesion molecules, and several enzymes associated with immune response and metabolism [29]. Previous evidence suggests that serine proteases found in the venom of *H. metabus* induces macrophage accumulation [17]. Nevertheless, recent data sustains that succinate and 2-hydroxyglutaric acid modulate macrophage function [30]. Specifically, 2-hydroxyglutaric acid induces the expression of pro-inflammatory IL-1β mediated by the activation of the transcription factor HIF-1α [30]. Ethanol is associated with skin irritation or contact dermatitis, especially in humans with an aldehyde dehydrogenase (ALDH) deficiency, whereas the contact of ethanol with healthy skin cells may result in localized erythema [31]. The intraperitoneal injection of ethanol caused an evident inflammation or fibrosis during the first 2 weeks in rats [32].

Glutamic acid is the main excitatory neurotransmitter used by primary afferent synapses and neurons in the spinal cord dorsal horn [33]. This amino acid and its neuronal receptors are located in areas of the brain, spinal cord, and periphery that are involved in pain sensation and transmission [33]. It is known that the intracellular concentration of glutamate in neurons is in the millimolar range [34]. Then, an excess in the extracellular concentration of glutamate may lead to excitotoxicity in vitro and in vivo via the overactivation of ionotropic glutamate receptors [34]. Since the concentrations of glutamate in the venom of *H. continua* is 6000 times more abundant than that required for neuronal communication, its possible toxicity at a local level cannot be discarded. Phosphocholine can be merged to specific proteins of nematodes and human placenta as a posttranslational modification to suppress an immune response by their hosts [35]. This evidence opens new and exciting research lines to demonstrate that phosphocholine from venom may reduce the immune response in human cells.

To the best of our knowledge, little information is available on the potential toxicity of alanine, glycine, and phenylalanine in individuals with normal metabolism. Nonetheless, the latest evidence sustains that glycine exerts moderate anti-inflammatory activity in murine models [36]. Unexpectedly, alanine intake stimulates the production of inflammatory cytokine IL-6 during running exercise [37]. The toxic effects of histidine are in a similar status than that already reported for alanine and glycine; however, the amino acid is considered an inflammatory marker of oxidative stress in obese persons [38]. The potential toxicity of proline is controversial, but it has been proven that this amino acid stimulates oxidative stress in the cerebral cortex of rats [39].

The role of polyamines as harmful compounds has been deeply screened since the presence of putrescine and cadaverine in some foods is latent and represents a threat for consumers [40,41]. The exogenous application of both amines in rabbits (10 mg mL^−1^) causes an inflammatory reaction by expressing tumor necrosis factor-alpha (TNF-α), interleukin-1 (IL-1), and interleukin-1 (IL-6) [41]. Remarkably, the amounts of both amines found in the venom of *H. continua* were ten-fold higher than those reported for inducing a systemic response (~100 mg L^−1^), and coincidently, previous works based in ^1^H NMR profiling report the presence of at least one biogenic amine in the venoms or venom-storing organs of some insects [23,24].

Acetoin is extensively used as a flavor in food products and causes damage to DNA and proteins [42]. This effect is strongly related to the reactivity of its keto group. Proteome profiling revealed that acetoin produced an evident stress associated with changes in the endogenous levels of lipid metabolic proteins [42]. Additionally, this compound promoted substantial fluctuations in fatty acid composition, with massive accumulation of cycC19:0 cyclopropane fatty acid in *Lactobacillus lactis* [42]. Further investigation is required to determine similar effects in human cells. Urocanic acid possesses well-known immunosuppressive properties which can induce the intracellular production of ROS-triggering oxidative DNA damage [43]. Controversial results have been gained on the possible use of this molecule as a “natural sunscreen” and as a mediator of photoimmunosuppression [43].

Finally, catechol and trigonelline were found in small quantities in the venom of *H. continua*; however, these compounds are well known to possess direct or indirect antioxidant activity [44,45]. Despite the potential benefits of these substances, it is known that elevated concentrations of antioxidants can actually exert prooxidant effects [46].

According to our results, the venom from *H. continua* contains molecules that act at different biochemical levels, probably causing a synergy to enhance inflammatory- and pain-inducing responses.

## 4. Conclusions

Nineteen small molecules were determined in the intact venom of *H. continua*. Most of these molecules possess biological activity as pain-inducing toxins, inducers of inflammatory response, or as natural immunosuppressors. To the best of our knowledge, this is the first investigation based on an NMR approach for characterizing the small molecule content of venom from a caterpillar associated with frequent cases of lepidopterism.

## 5. Materials and Methods

### 5.1. Collection and Identification of Hylesia continua

Larvae of *Hylesia continua* were collected in maize plots located in Yaonáhuac, Puebla, México (19°56′55″ N 97°26′26″ W; 1997 masl) during the raining season of 2021 (June–August 2021). Sixty larvae were transported to the laboratory in plastic containers covered with air flow tops. The larvae were fed ad libitum with maize leaves under 12 h light and 12 h darkness during the time of analysis. The morphological characteristics were contrasted with those of dichotomous keys for Saturniidae family and *Hylesia* genus [20,47]. The molecular identification of *H. continua* was achieved via DNA extraction of samples followed by the amplification of cytochrome oxidase subunit 1 gene (COI), using the oligonucleotides LCO1490 (GGTCAACAAATCATAAAGATATTGG) and HC02198 (TAAACTTCAGGGTGACCAAAAAATCA) [48]. The run conditions were 94 °C for 3 min initial denaturation, 40 cycles consisting of 94 °C denaturation, 56 °C for 30 s annealing, and 72 °C for 1 min 30 s polymerization. Amplicons were visualized in 1% agarose gel electrophoresis and purified using the gene elute PCR cleanup kit (Sigma-Aldrich Co., St. Louis, MO, USA) for further sequencing through internal services at the Centro de Biotecnología Genómica-IPN, using an ABI PRISM3130 (Applied Biosystems; Waltham, MA, USA). The sequences were compared with those stored in the National Center for Biotechnology Information using the BLAST approach.

### 5.2. Venom Extraction and Sample Preparation

The setae from 50 larvae of *H. continua* were slightly shaved with sterile steel scissors and the emerging endogenous fluid was dropped in sterile amber glass vials of 1.5 mL (Figure 1C). A total volume of 100 µL was finally obtained. The larvae were returned to the original site of collection after venom extraction. Fifty microliters of pure venom were resuspended deuterium oxide (D_2_O, 99.9 atom % D) which was purchased from Cambridge Isotope Laboratories, Inc. (Tewksbury, MA, USA). For ^1^H-NMR analysis, 3-(trimethylsilyl)-1-propanesulfonic acid sodium salt (Sigma-Aldrich Co., St. Louis, MO, USA; TSP, 97%) was used as an internal standard. Ethylenediaminetetraacetic acid (EDTA) and sodium azide (NaN_3_) (Merck^TM^, Darmstadt, Germany) were also added to the samples. NaOH and HCl (Sigma-Aldrich Co., St. Louis, MO, US.) were used to adjust pH levels.

### 5.3. NMR Analysis

Nuclear Magnetic Resonance spectra were obtained in a Bruker 17.6 T (750 MHz) spectrometer equipped with a TCI cryoprobe and a console AVANCE III coupled to TOPSPIN 3.7 software (Bruker Biospin, Rheinstetten, Germany). The temperature was kept at 298 °K. The 1D spectra were obtained using a NOESYPRESAT pulse sequence (noesypr1d) consisting of 10 s relaxation delay and 0.1 s mixing time. The free induction decay was obtained with 65 K data, 256 scans with a spectral width of 10 ppm. The acquisition time was 2.18 s and receiver gain of 4. The spectra were processed by Fourier transform and automatically put in normal phase. The baseline was manually corrected using TOPSPIN 3.7 software. All spectra were calibrated using the singlet of the methyl group from TSP, and the chemical shift (δ) was fixed at 0.00 ppm. The 2D ^1^H-^1^H COSY spectra were obtained using a cosygpppqf pulse sequence using a recycle delay of 2.0 s, and a gradient pulse length of 1.5 ms. The spectral width was set at 10.0 ppm in both dimensions, and 128 increases were recovered with 1024 points for both dimensions. The number of scans was 16, and the receiver gain was 30.

The ^1^H-^13^C HSQC spectra were obtained with a hsqcedetgpsp pulse sequence using a spectral window of 10.0 ppm for ^1^H and 180.0 ppm for ^13^C with gradient selection in anti-echo mode. A total of 64 scans were performed with 2048 points per increase. The recycle delay was 1.5 s, and the acquisition time was 0.04 s, whereas the coupling constant was ^1^*J*_CH_ = 145 Hz.

The spectra of NMR 2D ^1^H-^13^C HMBC were acquired with the hmbcetgpl3nd pulse sequence containing 256 increases with 32 scans and 2048 points per increase. The spectral width was 10.0 ppm for ^1^H and 220 ppm for ^13^C with an acquisition time of 0.18 s and a recycle delay of 2.0 s. The coupling constants were ^1^*J*_CH_ = 145 Hz and ^n^*J*_CH_ = 10 Hz.

Metabolite concentration was calculated using the qNMR method previously reported by Villa-Ruano et al. [49].

## Figures and Tables

**Table 1:** Small molecules detected in the venom from *Hylesia continua* via ^1^H NMR and its corroboration via two-dimensional NMR.

	Metabolite	Experimental Data				Confirmation	HMDB Data				Assignment
		δ ^1^H (ppm)	Multiplicity	*J* (Hz)	δ ^13^C (ppm)		δ ^1^H (ppm)	Multiplicity	*J* (Hz)	δ ^13^C (ppm)	
**Amino acids**											
1	Alanine	1.47 3.76 -	d q -	7.2 7.2 -	18.67 53.06 176.38	^1^H, COSY, HSQC, HMBC	1.46 3.77 -	d q -	7.1 7.20 -	19.03 53.55 177.35	CH_3_-6 CH_2_-4 C=O
2	Glycine	3.55 -	s -	- -	44.03 172 64	^1^H, HSQC, HMBC	3.54 -	s -	- -	44.30 173.59	CH_2_-4 C=O
3	Glutamic acid	2.04 2.12 2.35 - -	m m m - -	- - - - -	29.69 29.69 36.07 174.87 181.05	^1^H, COSY HSQC	- 2.08 2.34 - -	m m m - -	- - - - -	29.82 29.82 36.35 175.82 182.43	CH_2_-3 CH_2_-3 CH_2_-2 C=O C=O
4	Histidine	7.17 8.11	d d	- -	119.73 138.19	^1^H, HSQC	7.09 7.90	d d	0.6 1.1	119.99 138.36	CH-5 CH-2
5	Phenylalanine	7.32 7.36 7.41	d m m	7.5 - -	131.32 129.36 130.92	^1^H, HSQC	7.32 7.36 7.42	d m m	6.9 - -	132.11 130.42 131.81	CH-6, 2 CH-4 CH-3, 5
6	Proline	3,32 3.41 4.12	m m m	- - -	48.67 48.67 63.57	^1^H, HSQC	3.33 3.41 4.12	dt dt dd	14.0, 7.1 11.6, 7.0 8.8, 6.4	48.95 48.95 64.03	CH_2_-2 CH_2_-2 CH-5
**Organic acids**											
7	Acetic acid	1.91 -	s -	- -	25.86 180.81	^1^H, HSQC, HMBC	1.91 -	s -	- -	26.08 176.55	CH_3_-4 C=O
8	Formic acid	8.44	s	-	-	^1^H	8.44	s	-	172.41	CH-2
9	Lactic acid	1.32 4.10 -	d q -	6.9 7.0 -	22.71 71.15 183.46	^1^H, COSY, HSQC, HMBC	1.32 4.10	d q -	7.0 7.0 -	22.90 71.37 185.08	CH_3_-3 CH_2_-2 C=O
10	Succinic acid	2.40	s	-	36.61	^1^H, HSQC	2.39	s	-	36.82	CH_2_-4, 5
11	2-Hydroxyglutaric acid	1.84 1.99 2.29 4.02 -	m m m m -	- - - - -	33.84 33,84 36.24 71.39 182.57	^1^H, HSQC, HMBC	1.83 1.98 2.25 4.01 -	m m m m -	- - - - -	33.66 33.74 36.02 74.80 183.83	CH_2_-2 CH_2_-2 CH_2_-3 CH-1 C=O
12	Urocanic acid	6.41 7.28 7.43	d d s	16.0 16.0 -	- - -	^1^H, COSY, HSQC	6.38 7.29 7.38	d d m	16.0 16.1 -	124.55 133.42 123.94	CH-4 CH-3 CH-2
**Other compounds**											
13	Acetoin	1.36 2.22	d s	7.2 -	- -	^1^H	1.37 2.21	d s	7.2 -	20.91 27.55	CH_3_-3 CH_3_-6
14	Cadaverine	1.41 1.70 3.00	m m m	- - -	- 29.05 41.36	^1^H, COSY, HSQC	1.34 1.49 2.68	m m m	- - -	25.90 32.86 42.82	CH_2_-3 CH_2_-2 CH_2_-1
15	Catechol	6.85 6.93	m m	- -	123.88 119.15	^1^H, COSY, HSQC	6.88 6.96	m m	- -	123.81 119.12	CH-2 CH-1
16	O-Phosphocholine	3.21 3.60 4.18	s m m	- - -	56.61 68.85 60.90	^1^H, COSY, HSQC	3.19 3.57 4.15	s m m	- - -	56.52 68.90 60.60	CH_3_-5, 6, 7 CH_2_-2 CH_2_-3
17	Ethanol	1.17 3.64	t q	7.1 7.1	19.46 59.93	^1^H, COSY, HSQC	1.17 3.65	t q	7.1 7.1	19.59 60.30	CH_3_-3 CH_2_-2
18	Putrescine	1.75 3,03	m m	- -	26.53 40.59	^1^H, COSY, HSQC	1.75	m m	- -	26.72 41.73	CH_2_-2 CH_2_-1
19	Trigonelline	4.43 8.06 8.82 8.83 9.11	s m m m s	- - - - -	- - - - -	^1^H	4.43 8.07 8.78 8.87 9.16	s m m m s	- - - - -	51.06 130.09 148.71 147.51 148.38	CH_3_-9 CH-4 CH-5 CH-3 CH-1

s = singlet; d = doublet; t = triplet; dd = doublet of doublets; m = multiplet.

**Table 2:** Concentration of small molecules dissolved in the venom from *H. continua*.

	Metabolite	Concentration (mM)
1	Alanine	2.7708
2	Glycine	3.2829
3	Glutamic acid	5.4098
4	Histidine	1.3714
5	Phenylalanine	0.0755
6	Proline	1.2543
7	Acetic acid	28.1704
8	Formic acid	0.4487
9	Lactic acid	6.4347
10	Succinic acid	9.7295
11	2-Hydroxyglutaric acid	6.4660
12	Urocanic acid	0.1029
13	Acetoin	0.2760
14	Cadaverine	1.3077
15	Catechol	0.1029
16	O-Phosphocholine	3.0128
17	Ethanol	9.1453
18	Putrescine	1.5733
19	Trigonelline	0.0259

## Data Availability

Not applicable.

## Supplementary Materials

The following supporting information can be downloaded at: https://www.mdpi.com/article/10.3390/toxins15020101/s1, Sequence S1: Mitochondrial DNA sequence of the cytochrome oxidase subunit 1 (COI) gene from *Hylesia continua* amplified with the oligonucleotide LCO1490 reported by Folmer et al. [48]. Sequence S2: Mitochondrial DNA sequence of the cytochrome oxidase subunit 1 (COI) gene from *Hylesia continua* amplified with the oligonucleotide HC02198 reported by Folmer et al. [48]. Figure S1: Regions of the ^1^H NMR spectrum of the venom from *Hylesia continua* collected in Yaonáhuac, Puebla, Mexico.
