# Supplementary material for: 1H NMR Profiling of the Venom from Hylesia continua: Implications of Small Molecules for Lepidopterism
Source: Toxins (Basel). 2023 Jan 20;15(2):101. doi: 10.3390/toxins15020101 (PMC9962855; doi:10.3390/toxins15020101)
Supplement: Supplementary file 1 [file toxins-15-00101-s001.zip › toxins-2119072-supplementary.pdf]

ACAAAANTTATGATATCGTATGTCCTCCCTCCCTTCCTTGCAGGGTCAAAAAAAGAAGTATTTAAA
TTACGATCTGTTAATAATTATTGTAATAGCTCCTGCTAAAACAGGAAGAGAAAGAAGAAGAAGAAA
AGCTGTGATTCCTACTGATCAAACGAATAAAGGTATTTGATCAAAAGATATATTATTTAATCGTAT
ATTAATAATTGTTGTAATAAAATTAATAGCTCCTAAAATTGAGGAAATTCCAGCTAAATGAAGAGA
GAAAATAGCTAAATCTACTGAAGATCCTCCGTGGGCAATATTAGAGGATAAAGGAGGGTAAACTGT
TCAACCTGTTCCAGCTCCATTTTCTACAATTCTTCTTGAGATTAATAAAGTTAAAGAAGGAGGCAA
TAGCCAAAATCTTATATTATTTATTCGAGGGAAGGCTATATCAGGGGCTCCTAATATTAATGGAAC
TAATCAATTTCCAAACCCTCCAATTATAATAGGCATAACTATAAAAAAAATTATAATAAAAGCATG
AGCAGTTACAATTGTATTATAAATTTGATCATCTCCAATTAAAGATCCAGGGGTTCCTAATTCAGC
TCGAATTAGTAATCTTAAGGAGGCTCCGACTATTCCAGCTCAAATTCCAAAATTATTATATAAAGG
TTCCAATATCTTTATGATTTGTTGA

Sequence S1. Mitochondrial DNA sequence of the cytochrome oxidase subunit 1 (COI) gene from *Hylesia continua* amplified with the oligonucleotide LCO1490 reported by Folmer et al. [48].

GTCGATCCAAATACTCTGCGATTTAAGCTGGATAGTCGGAACCTCTTTAAGATTACTAATTCGAGC
TGAATTATGGAACCCCGGGATCTTTAATTGGAGATGATCAAATTTATAATACAATTGTAACTGCTC
ATGCTTTTATTATAATTTTTTTTATAGTTATGCCTATTATAATTGGAGGGGTTGGAAATTTAATAG
TTCCATTAATAATAGGAGGCCCTTAAATAGCCTTCGCCTCGCCAATTAATATAAGGAAGGGAGAAA
GTCATTGCTTCTTTAGTTTTTTCTGAGGGGGGGGAGAAAGGGGGGAGGAGGGGGGGGGGGGTGAGG
GGGGGGGGAGGGCGGTTGTGTTTTACATAAAGGAGGGCGCCGGGGGGCCGG

Sequence S2. Mitochondrial DNA sequence of the cytochrome oxidase subunit 1 (COI) gene from *Hylesia continua* amplified with the oligonucleotide HC02198 reported by Folmer et al. [48].

toxins

MDPI

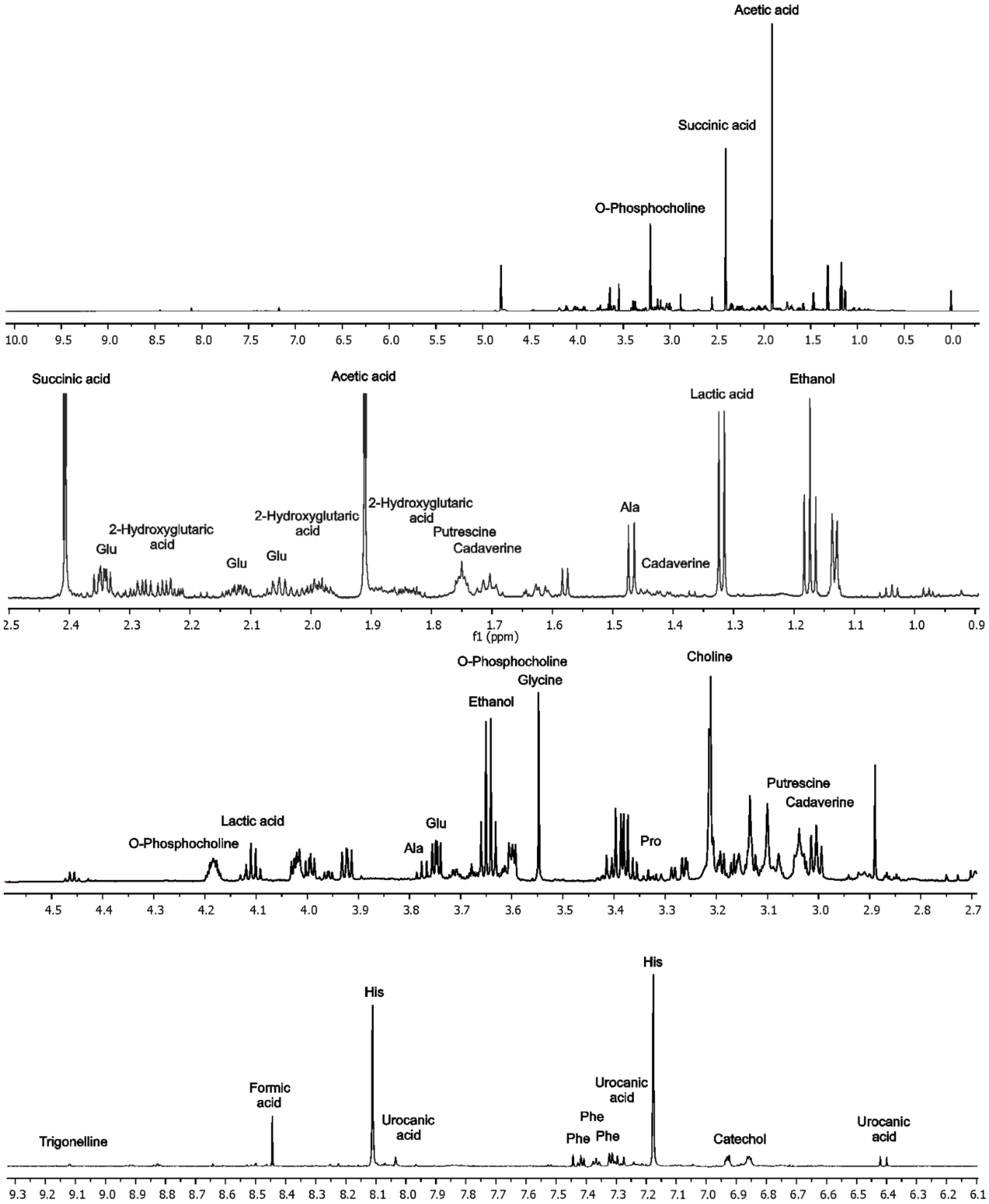

Figure S1. Regions of the ^{1}H NMR spectrum of the venom from *Hylesia continua* collected in Yaonáhuac Puebla-Mexico.
